# Administrative logic of grassroots community epidemic prevention from the perspective of attention allocation: evidence from Wuhan City

**DOI:** 10.3389/fpubh.2025.1604293

**Published:** 2025-06-26

**Authors:** Yanhua Zhang, Zuying Xu, Jiaxi Xu

**Affiliations:** ^1^School of Public Policy & Management, China University of Mining and Technology, Xuzhou, China; ^2^School of Economics and Management, Huaibei Normal University, Huaibei, China; ^3^School of Political Science & Public Administration, Wuhan University, Wuhan, China

**Keywords:** grassroots government, attention allocation, attention intensity, attention spans, epidemic prevention, emergency management

## Abstract

**Background:**

Chinese grassroots governments utilize fewer administrative resources to carry out tasks assigned by higher levels of government. They have refined their attention allocation into two dimensions: intensity and span, and have developed different action models for routine and non-routine tasks. This management style is becoming increasingly common in Chinese grassroots government operations.

**Methods:**

This paper presented a multiple case study of policy practices in Wuhan City, Hubei Province. The study analyzed the attention allocation practices of China’s grassroots government in high-pressure situations, particularly during the COVID-19 outbreak.

**Results:**

Grassroots governments can conserve attention resources by allocating attention efficiently and flexibly to deal with the dilemma of ‘too many tasks with too little power’. We summarized three models of coping by grassroots governments: (1) Attention allocation model in routine tasks; (2) Attention model in non-routine tasks; and (3) Routinization model through attention diversion.

**Conclusion:**

This paper presented a framework for explaining grassroots government behavior from an attention allocation perspective. We also identified some limitations of this model, both as a complement to attention allocation research and for a better understanding of grassroots government behavior in China.

## 1 Introduction

In early 2020, COVID-19 broke out in Wuhan and quickly spread throughout the country. The grassroots government, as the main body in charge of the epidemic prevention, were under tremendous pressure (1). As the destination of public policy implementation in China, public policies are created by the higher-level of governments but are implemented by the lower-level of governments (2). Grassroots governments have to deal with the almost comprehensive tasks of governance, though they have fewer human and administrative resources. In particular, in the major emergency, the fragmented responses of local authorities and non-state actors have played an important role (3). Accordingly, grassroots governments have limited resources for attention, which is reflected in the limitations of their functional competence (4), insufficient material resources (5), and the impact of strong accountability pressures (6).

Many studies found that the rational allocation of attention was a central tenet of China’s grassroots government in taking on these tasks. In China, various tasks were assigned and pressured from one level to another, and lower-level government functions were passively accepted (7). In response, local policy activism and experimentation were not only allowed but also largely encouraged so long as they were apolitical and able to generate innovative solutions for policy problems (8, 9). Through objective political practices such as meetings and policies, we can identify the unequal distribution of government attention in practice. In addition, specific behaviors of grassroots government, such as the degree of resource commitment to a particular task, continuity of behavior, implementation of policies and other behavioral-level moves, may also reflect the allocation of grassroots government attention.

As a model of policy and administrative, attention allocation is not only an activity of grassroots governments affected by various factors, but also reflects various relationships between central or higher-level governments and grassroots governments. So, in a general sense, it exists in the bureaucratic systems of the vast majority of countries. The process of policy implementation is not only a technical or procedural issue, but also reflects a political aspect, reflecting the power relationship between the central, higher-level, and grassroots governments. In the West, local autonomy is not a modern concept, but has a profound historical origin (10). This tradition emphasizes the self-management and decision-making of local communities, which has been developed and practiced for a long time in many European countries. Local governments have significant autonomy in areas such as education, transportation, and the environment (11). This means that local governments can formulate and implement corresponding policies based on local conditions and needs, without relying entirely on instructions from the central government. It makes local governments largely independent of the central government.

However, the complexity, suddenness, and spillover nature of social affairs and issues have changed this relationship. Andert and Nagel analyzed the controversy surrounding the tram project in Tübingen, Germany, revealing how the game between 19 stakeholders (including environmental organizations, transportation companies, and community groups) led to a governance deadlock in the “green demonstration city,” confirming the dissolving effect of the complexity of social issues on local autonomy (12). Ruijer et al. demonstrated through research conducted by the Dutch Living Laboratory that open data work, through a triple mechanism of “intermediary agency coordination data problem definition public participation,” can increase local government decision-making response speed, but requires the reconstruction of traditional hierarchical power structures (13). The financial impact of the central government on local governments cannot be ignored. A study found that excessive intervention by the central government of EU countries in local finances can lead to the failure of carbon price signals and weaken the efficiency of grassroots policy implementation (14). When the central government cuts social welfare spending, grassroots governments are forced to balance the budget by raising taxes or reducing public services, leading to policy implementation deviating from established goals (15). A study on performance contracts for local governments in New Zealand showed that setting quantitative indicators by the central government increased policy implementation efficiency by 19%, but led to 41% of grassroots officials choosing the “optimal solution of indicators” rather than actual demand orientation (16).

Of course, the COVID-19 also triggered a surge of interest in reviewing public sector responses. The epidemic exposed the inadequacy of government response at all levels and revealed the vulnerability of healthcare, insurance, and public sectors. A study found that Italy faced a “dual decision-making” problem between central and local governments in the early stages of the 2020 pandemic. The central government attempted to unify the blockade policy, but local governments (such as Lombardy) adjusted measures under the pretext of “local autonomy,” resulting in policy fragmentation (17). A case study of Florida in the United States showed that the federal government’s mask mandate and vaccination policies had been resisted by state governments on the grounds of “violating state rights.” The governor even signed an executive order prohibiting local governments from implementing epidemic prevention restrictions, forming a three-level confrontation of “federal state city” (18).

The allocation of resources during the epidemic has also caused tension between the central government and local governments. For example, in the allocation of ventilators in Belgium in March 2020, the central government allocated resources based on the proportion of the population, but the severely affected Walloon region believed that the actual infection rate was not taken into account, resulting in local governments purchasing through EU channels on their own, causing the failure of the national reserve system (19). The UK National Audit Office report found that the central government initially monopolized the procurement rights of personal protective equipment (PPE), but the distribution efficiency was low. Manchester and other local governments were forced to establish parallel supply chains, leading to price hikes and repeated purchases, which ultimately prolonged the shortage of PPE in Britain for at least 3 months (20). The introduction of digital technology into epidemic prevention policies has not been smooth sailing. According to data from the Paris Regional Health Department, the Central Epidemic Command Center took an average of 52 h to process local reported data, resulting in an “information vacuum period” in Lyon and other areas. Local governments therefore established alternative monitoring indicators, but the differences in indicator calibers caused distortion in the national epidemic map (21). During the development of Germany’s COVID-19 early warning APP, the federal government and the state government had differences on data standards and privacy protection rules, and Bavaria even suspended access to the national contact tracking system. This contradiction is rooted in the German tradition of “cultural federalism,” and there is a natural vigilance among localities toward centralized data (22).

These studies have focused on the contradictions between the central and local governments in western countries in the process of COVID-19 prevention and control, which are mainly reflected in the allocation of policy implementation rights, resource allocation conflicts, information coordination mechanism defects, etc. These studies reveal that sudden public health emergencies have magnified the shortcomings in the allocation of rights and responsibilities in the existing governance system, and the efficiency of crisis response depends on the dynamic balance between central coordination and local flexibility. The COVID-19 challenged local and national capacities to prepare and respond. It provided a renewed prospect for solidarity within the country.

During COVID-19, grassroots governments carried out tasks assigned by higher-level governments through various attention allocation strategies. By examining the epidemic prevention practices of grassroots governments in Wuhan City, Hubei Province, this paper analyzed the impact of attention allocation on government behavior by refining it into two dimensions, namely the attention intensity and attention span. At the same time, situations were created in which grassroots governments respond to three types of tasks: ‘routine tasks’, ‘non-routine tasks’, and ‘routinization of non-routine tasks’, in order to better explain the logic and effectiveness of grassroots governments’ attention allocation.

## 2 Literature review

Herbert A. Simon, in proposing a model of finite rationality, argued that the scarcity of attention proves that decision-makers are not omniscient (23). Jones (24) built on this by modeling decision-making in democratic politics, distinguishing between decision-makers’ attention and their preferences. Baumgartner et al. (25) explained that people’s limited attention span was a very important factor in influencing political agendas. How to solve the problem of making the best decisions for each individual given the information available, and how to design efficient and flexible government institutions, was a major issue in the social sciences (26). Like individuals, government agencies suffered from the problem of attention scarcity when dealing with information (27). Bureaucracies in the political process, with conflicting goals and facing severe attention scarcity constraints, had to adopt certain simplified ways of filtering information in order to achieve these goals. Attention allocation had received much attention in the field of management and was a key factor in government decision making (28).

Since the beginning of the post-Mao reforms, the China’s grassroots government reforms developed in response to socio-political changes (29). Grassroots governments were an extension of the central government and must act in accordance with requirements. This was reflected in the ability of the Party Committee to selectively and efficiently priorities the implementation of certain policies and the identification of issues to be addressed, as well as to control the development of certain key areas and the appointment and dismissal of officials (30). At present, effective control by the higher-level government over the lower-level government was exercised mainly through evaluation. This was both supervision of delegated authority and effective monitoring of the management of the agent (lower-level government). It not only strengthened the ability of higher-level government to implement governance, effectively resolved the conflict between jurisdiction and governance, but also effectively compensated for the limited attention of higher-level government itself (31). However, China’s grassroots government also had full autonomy. For the government at the lower-level, because of attention scarcity, the main question that grassroots governments think about is how to use their limited attention resources to solve the key tasks.

Cheng and Yang (32) studied the allocation and evolution of the Chinese government’s attention to the power industry based on 2,230 policy documents. Meng and Fan (33) explored the punctuations and diversity in attention allocation within China’s national e-government issue from 2001 to 2018. Hu et al. (34) measured the change in attention allocation of the Central Committee of the CPC’s land policy based on a comparative analysis of the Central No. 1 document. In summary, in the Chinese discourse context, the research on the government’s attention allocation had included many aspects, but the current research was mostly based on the analysis using the Dirichlet allocation topic model, which lacked the logical exploration of the government’s attention allocation, and lacked the analysis of the intrinsic reasons for the changes in the attention allocation.

Obviously, attention allocation also involves issues or influencing factors such as the relationship between local and central government, public attitudes and pressure from the public. After tracking the trend of European municipal mergers, Van Houwelingen concluded that local autonomy in Europe has, on average, increased since 1990 and has decreased (a little) since 2009. Residents of small cities were relatively more willing to participate in local affairs (35). Nabatchi et al. (36) criticized that traditional bureaucratic systems have a dual failure in addressing “wicked problems” such as climate change. Because it cannot maintain political neutrality and was difficult to integrate cross domain resources. It was also necessary to redefine the scope of local autonomy. Strebel and Kübler (37) found based on survey data from 12 Western European countries that the majority of citizens support strengthening local autonomy, but hold reservations about inter-local cooperation. They argued that citizens’ attitudes toward local autonomy and inter-local cooperation are a function of their behavioral, emotional and ideological connection to the local (37). Under such circumstances, public attention undoubtedly influenced the allocation of government attention in different forms and to varying degrees. Jennings and John found that there was a long-term equilibrium state between public opinions and government attention, and they also discussed the responsiveness between policies and opinions (38). Xia and Shen (39) studied the dynamic relationship between public opinions and government attention after the return of Hong Kong to China, providing cases of non-Western societies. Aksoy et al. (40) analyzed how public attention (such as through Google search volume) during the COVID-19 period influenced the speed at which the government implemented non-pharmaceutical interventions, demonstrating the impact of public attention on the response time of policies. Bi et al. (41) discussed how the disclosure of food safety and environmental protection information affects government supervision through public pressure. It was a very good case analysis of the transformation of public demands into policy implementation.

As described by Xu et al. (42), “the government attention has become a scarce resource, and appropriate allocation is a necessary condition for obtaining effective safety information and improving safety management.” The study of security management was enriched by examining the allocation of government attention. We found that existing research lacked attention to the allocation of government attention in crisis situations. There was ambiguity in examining the logic through which attention affected government behavior. At the same time, existing research lacked attention to the attention allocation of Chinese grassroots governments. We believed that the following three concepts deserved attention to address the above issues.

### 2.1 Attention allocation in organizations

Attention is the ability to focus one’s cognitive abilities on a particular objection while ignoring others, the essence of which is selectivity (43). Attention allocation is defined as our brain’s ability to attend to two different stimuli simultaneously (44, 45), while responding to multiple demands around us. Attention allocation is influenced by passive attention (also known as non-casual attention) and active attention (also known as casual attention), and are a type of attention that allows us to process different sources of information simultaneously and successfully perform multiple tasks at once.

With the development of attention allocation theory, scholars have used it to explain government behavior (46). The government views attention allocation as a concept that can be divided into attention at the cognitive and behavioral levels (47). Government attention allocation refers to the decision-making behavior of officials in allocating their attentional inputs to issues that may rise to the level of a policy issue. Attention allocation is the starting point of the decision-making process. In the context of limited attention resources, decision-makers cannot deal with multiple public services simultaneously. “It is essential that officials gauge the task’s priority according to its importance, so as to allocate attention resources efficiently” (48). Attention allocation is therefore the starting point of the decision-making process.

Generally speaking, bureaucratic control depends not only on how information is obtained, but also on how information is processed, that is, how attention is dispersed in specific fields or focused on specific issues (49). Under the Chinese system, the policy orientation of higher-level governments undoubtedly affects the policy priorities of local governments (50). For example, in the case where the central government has made environmental protection and resource conservation a national key policy, research has identified and quantified vocabulary related to the ecological environment in government work reports through word frequency analysis, in order to measure the level of concern of local governments for the environment (51). The attention of local governments and the allocation of policy resources largely reflect the priority and effectiveness of addressing social issues. The attention of local governments to environmental issues can improve the efficiency of environmental governance, especially in controlling air pollution (52). With the development of information technology, policy attention as a signal has predictability in guiding government actions related to e-government. After analyzing panel data from 333 prefecture level municipal governments in China, it was confirmed that policy attention can improve e-government performance (53).

This study acknowledges that attention investment is a critical factor influencing the task completion and governance performance of local governments. However, the attention investment of local governments varies when facing different tasks, particularly when sudden events occur, leading to a shift in focus, an aspect that has been under-researched in previous studies. Therefore, this study proposes a model for attention allocation in the context of both routine and extraordinary tasks faced by the government. Building on this, the study focuses on the attention allocation and shift in local governments during the COVID-19 pandemic.

### 2.2 Attention intensity

Attention intensity is a dimension of attention allocation that refers to the amount of resources allocated (54). If the government allocates more attention to a matter, it will allocate more resources to it. Attention intensity can be disturbed by a number of external factors, such as policy experts, the media, and focal events. Our discussion on ‘attention intensification’, ‘attention recession’ and ‘attention focus’ is based on the dimension of attention intensity.

### 2.3 Attention span

Attention span is the time for which the original intensity of attention is maintained after attentional resources have been allocated. The more stable the government’s attention to a matter, the longer the attention span (55). The formation and diversion of the attentional focus are part of the change in attentional intensity, while the duration of the attentional focus before diversion is part of the attentional breadth. Attentional breadth is determined by the stability of attentional strength, although there is no correlation between attentional strength and duration. Attention can be either strong and stable or weak and stable.

## 3 Materials and methods

### 3.1 Study design

Simona et al. (56) analyzed collaborative services within government and between agencies in emergency management and proposed a 3C model based on communication, coordination and cooperation. Ufua et al. (57) examined the distribution of government supplies in Lagos State, Nigeria and proposed a model for a boycott approach to effectively address the challenges in the current distribution process of supplies in Lagos State, the center of the COVID-19 epidemic in Nigeria. It can be seen that the study on government behavior in public health emergencies is a common practice in the academic world and also has a strong value for practical analysis.

As Chao et al. (58) described, the popularity of the case study design stemmed from its ability to provide the researcher with a deeper understanding of specific individuals, an identified problem, or a distinctive situation by closely studying the phenomenon in intensive and great depth. A review of typical cases can better analyze the logic of attention allocation in disaster response at the grassroots government.

The cases come from a multi-year survey of urban public safety conducted by the lead author’s institution. Since 2018, the School of Public Policy & Management of China University of Mining and Technology continuously organized students to conduct surveys and interviews on the public sense of security in 36 key cities in China, and wrote and published survey reports and interview reports. After the outbreak of the COVID-19, the school quickly took the sense of security of residents and the prevention and control practices of grassroots governments as the themes of the surveys. Wuhan, as one of the 36 key cities under investigation and as the place where the COVID-19 firstly emerged in China, the region with the most severe epidemic situation and the key area for prevention and control, a lot of relevant information and materials naturally became the best materials for research and, of course, the best resources that most intuitively reflected the attention allocation of grassroots governments during the pandemic period.

In the past 5 years, the school where the first author of this study is located has completed interviews and questionnaire surveys on the epidemic prevention and control in more than seven subdistricts and 30 communities in Wuhan. These became the direct and original materials for this study. On this basis, Jiaxi Xu, one of the authors of this paper, was once a key member of the survey team and continued to conduct follow-up visits to the key communities among them and supplement relevant information according to the needs of the research after entering Wuhan University for postgraduate study. The practical surveys carried out as mentioned above have formed the foundation of this study and supported the main materials required for the research.

This study will examine how the Chinese grassroots government undertakes multiple tasks assigned by higher-levels through attention allocation under the constraints of limited attention resources, thus constructing the administrative logic of the Chinese grassroots government under the perspective of attention allocation.

In the surveys and studies, we selected subdistricts and communities as the basic units for observation. In China, the subdistrict is the grassroots unit for urban management, while the community is the basic unit of residents’ lives. The subdistrict is the most basic unit in the hierarchy of the Chinese government. It not only undertakes the tasks of policy transformation and governance from higher-level governments but also bears the responsibility of reporting social concerns and social problems to higher-level governments. Although communities are positioned as self-governing organizations of urban residents in the institutional sense, they are actually doing the work of subdistricts, yet focusing more on the micro aspects of residents’ lives. Communities will pay attention to the specific needs of residents, such as environmental improvement, cultural activities, neighborhood relations and so on, and meet these needs by organizing various activities and providing services. Together, they form the cornerstone of urban governance and social services.

After COVID-19 broke out and spread rapidly in Wuhan, subdistricts and communities became the front line of epidemic prevention and undertook direct and arduous epidemic prevention tasks. They were the main entities using attention and witnessed the micro-practices of government attention in special situations. In the face of the first outbreak and serious spread of COVID-19, the stories and responses of the grassroots government in Wuhan can provide direct reference experience for other local governments in China to do a good job in epidemic prevention. The facts also confirmed this view. Therefore, we chose the streets and communities of Wuhan as the sample for the case study.

### 3.2 Data collection

Control and prevention COVID-19 in Chinese cities was an example of grassroots governments allocating governance resources through attention allocation strategies, and was also a good way to observe the Chinese politics and government. COVID-19 had a high level of social attention and information disclosure, and had a direct and profound impact on economic and social development (59). This paper presented a multiple cases study of the grassroots government of Wuhan City during the COVID-19 epidemic. In addition to the information and data obtained from interviews and surveys over the years mentioned above, we analyzed data collected from public policy documents on government websites and news reports from the media, supplemented with daily observations. This study illustrated how grassroots governments in Wuhan prioritized tasks assigned by higher-level government and how this influenced their emergency response behaviors. We attempted to combine relevant theories and research results to innovate perspectives and ideas on allocation from attention allocation theory.

### 3.3 Cases presentation

#### 3.3.1 Attention allocation model in routine tasks

At the beginning of COVID-19, the management of *Subdistrict A* in Wuhan arranged different duties for the staff in the community to cope with a variety of tasks such as service, publicity, patrol, transport, help and supply (Table 1). At this time, during the COVID-19 prevention and control period, each kind of task had different importance to the grassroots government, and the attraction situation of attention was also different. The key tasks can get more attention resources tilted by the grassroots government. Under the organizational arrangement, about 40% of the people in subdistrict A served in the supply tasks, and 17% were engaged in helping special groups. According to the Wuhan COVID-19 Command Circular (No. 23), the division of labor arrangement in this community ended on 13 June 2020 and lasted for more than 5 months. Routine tasks had a long attention allocation span, and it was difficult for the grassroots government to divert its attention. Most of the government staff were deployed to take charge of routine tasks, which were key to the assessment of the higher-levels of government and had a direct impact on the life satisfaction and happiness of the residents.

**Table 1:** Assignment of responsibilities at the subdistrict A.

Work team	Numbers	Responsibilities
Team leader	1	Command the daily work of the team
Containment team	9	Infected area cordon duties and conduct daily patrols
Service team	7	Serving the community and publicizing government policies
Screening team	8	Identification of persons at risk
Special services team	7	Help for special groups
Supply team	35	Supplies for daily life, epidemic prevention
Volunteer team	7	Volunteering

#### 3.3.2 Attention allocation model in non-routine tasks

At the beginning of COVID-19, *Subdistrict B* in Wuhan received a huge amount of anti-epidemic supplies from all over the country. This subdistrict was faced with the extraordinary task of receiving and distributing these supplies due to their short-term and concentrated arrival. The subdistrict, which had only 13 regular employees, served a population of 5,635 permanent residents living in 2,751 households across 47 residential buildings. Following the donation, this subdistrict quickly mobilized party members, volunteers, and civil organizations to establish multiple teams for distributing materials. The task force was organized using the ‘1 + N’ model, with each group led by a government staff member and consisting of N party members or volunteers. The teams worked around the clock, with rationalized zones and divert rotations. In the process of distributing epidemic prevention materials, they also assisted residents in solving their daily life problems. The party secretary of subdistrict B provided guidance within the subdistrict and worked during his own rest time to assist in the distribution of epidemic prevention materials. The short-term organizational model of the grassroots government was effectively used to meet the challenges of the extraordinary task. The rapid concentration of attention and resources enabled the grassroots government to gather enough volunteers to distribute supplies, even in the face of a lack of manpower.

#### 3.3.3 Routinization model through attention diversion

Since the outbreak of COVID-19 at the end of 2019, a number of places had entered a state of emergency, including Wuhan, which launched a first-level response on 24 January and adjusted to a second-level response on 2 May, lasting 98 days. In *Subdistrict J* of Wuhan City, a series of supportive measures were introduced. Staff were sent into enterprises to understand the difficulties they were facing in production and business activities, and coordinated with various departments to solve these problems. The government officials met with enterprise leaders to gain insight into the district’s economic situation. Additionally, CPC members, volunteers, and staff from subdistrict J conducted sanitation campaigns to support the resumption of work and production in the area while maintaining effective epidemic prevention measures. The COVID-19 pandemic brought various industries in the city to a standstill, adversely affecting the economy. As the situation improved, the government’s attention gradually shifted from epidemic prevention and control to other routine tasks, such as economy.

On March 20th, J subdistrict held a coordination meeting for resuming work and production, and established a special team for resuming work and production led by the main leaders of the street office. Establish a joint “resumption of work and production inspection team” consisting of regional development, safety supervision, food and drug supervision, fire protection, industry and commerce, public security and other departments, and a “resumption of work and production service team for individual industrial and commercial households” with the community as the core.

According to the minutes of this meeting, the key tasks that need to be implemented are as follows. Promote the types, time, process, channels, and required materials of enterprises that can be declared, and display the application process for resuming work and production in the form of charts and graphs; Actively communicate with enterprises in the jurisdiction to determine the list of employees returning to Wuhan, and implement the district government’s “point-to-point” centralized transportation of out of town employees back to Wuhan; Conduct a survey on the situation of newly added enterprises resuming work, and adopt a combination of self-declaration by enterprises and proactive government services to assist enterprises, especially those above a certain scale, in applying for resumption of work and production; The responsibility lies with individuals entering the enterprise, without resorting to formalities, strictly inspecting the enterprise’s epidemic prevention measures, and strengthening the service awareness of the “shop assistant”; Thoroughly investigate the practical difficulties encountered by enterprises within the jurisdiction in production and operation, and implement policy incentives such as rent reduction and subsidies, emergency financing funds for small and micro enterprises, etc.

## 4 Results

The examples above demonstrated the various attention allocation strategies employed by grassroots governments when faced with stressful tasks. A framework needs to be constructed to analyze how such a selective attention allocation model is achieved at the grassroots government.

### 4.1 An explanatory framework for attention allocation

**Figure 1:**
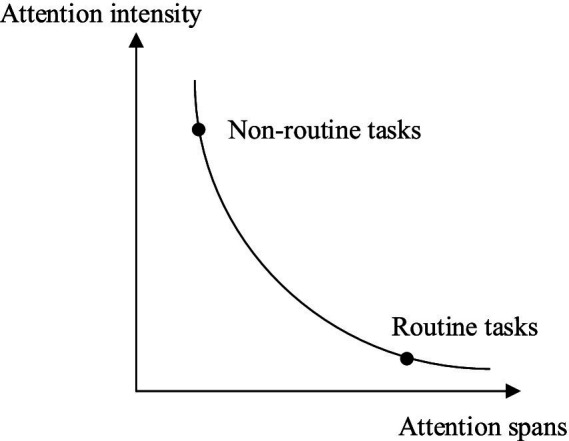
Attention allocation in routine and non-routine task scenarios.

Routine tasks are activities carried out by the government in a normal governance environment, in accordance with predetermined plans and objectives. These tasks are typically quantitative and diverse in nature (60). Task promotion is usually based on a bureaucracy. “There is generally a big gap between the routine tasks of governance and administration, on one hand, and the emergency, non-routine tasks that demand urgency in attention and action, on the other hand” (61). As shown in Figure 1, routine tasks generally only reap less attention intensity, but their duration can be longer. In routine tasks, the attention of grassroots governments is multi-level, but the intensity is not significant. The routine tasks of the government, such as public services, administrative processes, and budget execution, are the foundation for maintaining the operation of the national machinery and typically require stable human, financial, and time investments. These tasks often have rigid requirements and consume a significant amount of administrative resources. It is worth noting that grassroots governments have a traceable system and tacit understanding for resource and attention investment in completing routine tasks. The stockpiling of materials, information report development, joint notification and report release, epidemic announcements are typical tasks in epidemic prevention (62). These tasks are usually assigned by higher-level in a sectional system.

Based on an interview with community worker Zhang Ling, after the lockdown of Wuhan on January 23rd, she stayed in a safe hotel in her jurisdiction for the convenience of work and did not go home for more than 2 months. Including the secretary, there are a total of six people working for up to 12 h a day, sometimes even up to 15 h. Daily work includes screening of fever patients, coordination of medical treatment, community disinfection and sterilization, recruitment and management of volunteers, management of sunk cadres, distribution of living materials for residents, group purchase and distribution, donation of love materials, and assistance in solving residents’ difficulties. Until early February, as the epidemic continued, a group of volunteers had to be recruited through Civilized Wuhan. After screening, more than 90 cadres were added to support the sinking. These personnel have been trained, classified and assigned to their positions, and their responsibilities are in place. The volunteers responsible for community control work from 8 am to 8 pm, with two people guarding for 12 h and handing over shifts in the morning and evening to ensure 24-h community control. In addition, after the community is closed, residents’ living security will be uniformly arranged by the community. The community where Zhang Ling resides has over 3,000 households with diverse needs. Community workers and volunteers are responsible for purchasing grain and oil supplies, daily necessities, medicines, etc. for residents. During the specific delivery process, in order to ensure that residents can stay at home with peace of mind, epidemic prevention personnel need to deliver these materials to their doorstep, sometimes lifting tens of pounds upstairs.

The indicators for this type of work are clear, and the operation is relatively simple, providing greater stability and predictability. At the same time, this type of work involves fewer relevant interest groups, is a routine matter for grassroots governments, and conflicts are not prominent. Based on the above analysis, we propose *Proposition 1*.

*Proposition 1*: The grassroots government will carry out an attention allocation model in routine tasks, and allocate attention in the pattern of low intensity and high spans.

“Contingency and the need to develop new activities quickly make administrative coordination based on preplanning of routine tasks obviously difficult.” (63) Most of the emergency management activities are urgent non-routine tasks. Non-routine tasks are governance activities carried out by governance actors in a non-routine or suddenly changing governance environment. They possess a strong attention intensity but short attention spans. These tasks are generally oriented toward specific events and goals, as shown in Figure 1. The main driving mechanisms for non-routine tasks are campaign-style governance (64) and project-based governance (65). The main characteristics of these changes are cross-sectoral, cross-system, cross-regional, and suddenness. They will result in a diversion in the power structure and routine operation mode of grassroots governments, leading to a reconfiguration of the unbalanced power distribution.

According to the epidemic prevention and control log written by Peng Li, a community worker, the Zhiyuan Community where she works is a resettlement community rebuilt from the shantytown renovation of a state-owned enterprise. There are 3,124 households and more than 8,000 people in total. At the toughest moment of the battle to defend Wuhan in 2020, among the 9 community workers in this community, one was in another city and one was placed under home quarantine. The remaining 7 were all women, with the youngest born in 1992 and her child just 6 months old. These 7 women had been sticking to their posts all the time. They were responsible for taking people’s temperatures, conducting screening, carrying out community prevention and control work, delivering vegetables and medicines, collecting and distributing express deliveries, and visiting poor households to show care. They were busy from morning till night every day and did not go home for a single day.

Some typical tasks involved are as follows: every morning, after taking a series of preventive measures (such as wearing masks and spraying disinfectant alcohol), they would carry a small loudspeaker and play the knowledge about epidemic prevention and control in a loop around the community, ensuring that residents could hear the broadcast once every 30 min. In addition, the community would promptly post announcements about COVID-19, relevant bans and various prevention and control knowledge on the bulletin boards at the entrances of each community and at the entrances of each building every day, so that residents in the jurisdiction could learn about the real-time situation of the epidemic in a timely manner, enhance their awareness of self-protection and reduce unnecessary panic.

The attention of grassroots governments is characterized by high intensity and low persistence in the performance of specialized tasks, so we propose *Proposition 2*.

*Proposition 2*: In non-routine tasks grassroots governments engage in flexible attention diversion, with high intensity, low duration patterns of attention allocation.

**Figure 2:**
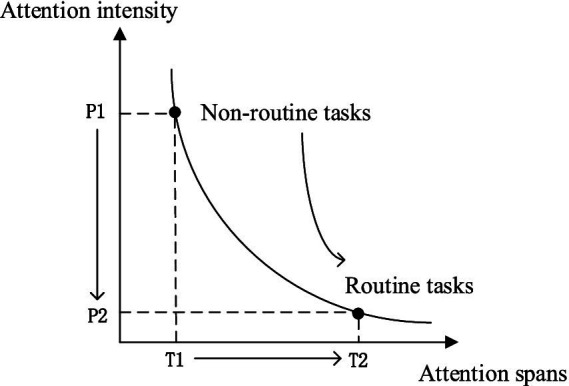
Attention changes in non-routine task scenarios.

The relationship between grassroots governments and their departments in China depends on the political mandate of the party organization, which is easy to assess due to characteristics such as the short duration of the mandate and the ease of measuring its effects. As a result, grassroots governments face greater institutional pressure in non-routine tasks, and those in charge of grassroots governments are easily influenced by the pressure to make decisions that avoid responsibility (66). The short-term nature of unconventional tasks, in turn, makes it easier to influence changes in government attention allocation. Figure 2 shows the changes in grassroots government attention when routine tasks are transformed into non-routine tasks. However, when emergencies (such as epidemics or economic crises) occur, the attention of grassroots governments will quickly shift to emergency management, and routine tasks may be simplified or delayed. As tasks are completed, attention quickly diverts and reallocates. Figure 2 shows that when non-routine tasks are overloaded onto routine tasks, resulting in a shift to regularized governance, the intensity of attention acquired decreases from P1 to P2. Meanwhile, because of the long duration of routine tasks, their attention spans increase from T1 to T2.

Taking into account the aforementioned analyses, we propose the integrative idea that local governments can address the challenge of having too many tasks and limited resources by conserving attention resources through efficient and adaptable allocation. This can be accomplished by allocating attention to routine tasks and diverting attention to non-routine tasks.

### 4.2 Organizational behavior under the influence of attention allocation

Grassroots governments can effectively undertake tasks assigned by higher-level governments through effective attention allocation and attention diversion. The administrative behavior of grassroots governments is ultimately reflected in changes in the allocation of attention. Therefore, this analysis focuses on how grassroots governments influence changes in government administrative behavior through attention allocation. Meanwhile, in response to COVID-19, some grassroots governments in Wuhan also experienced issues such as attention fatigue, distraction, and attention mismatch. Analyzing the causes of these problems can help China’s grassroots governments better cope with future challenges.

#### 4.2.1 Attention intensity affects resource allocation

Resource allocation is an important task in organizational management (67). In governmental organizations, resources generally include political resources (administrative power), human resources (civil servants), customer resources (including enterprises, institutions and citizens), financial resources, asset resources (property, vehicles, office equipment, etc.), and information resources (official documents, declarations, statistical data, etc.), all of which are limited.

When the government carries out the planning and allocation of resources, out of the consideration of improving administrative efficiency and reducing the administrative cost of the government, it will tilt the resources to the affairs that the government considers important and need resources (68). This process is known as attention allocation within the organization and is influenced by the government’s imperfectly rational decision-making (69). The government’s attention can be deduced from its allocation of resources. More resources indicate a higher level of attention, while fewer or inadequate resources indicate a lower level of attention.

#### 4.2.2 Attention span influences continuity of organizational behavior

Due to the scarcity of attentional resources, governments make decisions with limited rationality (70), often making decisions and taking actions before they have fully understood the information. The hierarchy rule is the initial stage in influencing the government’s attention allocation and actions. Once the hierarchy rule is adhered to, bureaucratic interests will dominate the government’s behavior. If external pressures are strong enough, the government’s attention will quickly divert externally, disregarding rules and bureaucratic interests. Factors have varying degrees of dominance at different points in time, and their strength affects their dominance.

During attention allocation, attention diverts and stabilizes as influencing factors change. However, sudden changes in influencing factors can cause diversion in the grassroots government’s attention. When these factors are stable, the government’s attention allocation is also stable, resulting in policies and behaviors with continuity. When one influencing factor is significantly stronger than others, the government’s focus diversion to that event. Therefore, attention span can be determined by the consistency of the government’s actions in a given task. The more consistent the behavior, the longer the attention span, and conversely, the shorter the attention span.

#### 4.2.3 Attention allocation affects the government’s implementation of higher-level mandates

In China’s pressure-based system, grassroots governments are both controlled by higher-levels of government and have a certain degree of autonomy, which means that they may engage in responsibility-avoidance behavior, which is closely related to the anti-function of the hierarchical system and the bureaucratic personality (71). As a result, there is a great opportunity for grassroots governments to man oeuvre in the details of work implementation, as well as passive and lax behavior toward tasks and misinterpretation of policies. The allocation of government resources and continuity of government behavior are affected by the intensity and spans of attention allocation discussed above. Poor task implementation results when the intensity and duration of attention allocation do not match the actual attention required for the task. In the specific process of task implementation, differences in attention allocation allow grass-roots governments to autonomously choose the order of policy implementation, formulate implementation programmers, and report on their work within the limits permitted by law and policy.

### 4.3 Problems with attention allocation in grassroots government

#### 4.3.1 Attention fatigue: the burden on grassroots governments

Attention fatigue is the result of an excess of attention resources, was operationally defined as decline in alerting, orienting, and executive attention performance (72). Attention resources are always limited, although governments can conserve these resources through flexible attention allocation strategies. Since the outbreak of COVID-19, urban and rural grassroots governments have become the first line of epidemic prevention and control (73). As the governance system bridging the state and society, they play a crucial role in managing the pandemic. In COVID-19 prevention and control, various routine and non-routine tasks have been refined at the grassroots level. These include mapping people’s movements, conducting household health surveillance, cleaning and disinfecting public places, and disseminating knowledge about epidemic prevention and control. This has resulted in an enormous workload for grassroots governments, with a shortage of manpower in the community and a significant burden on the grassroots governments.

#### 4.3.2 Distraction of attention: inefficiency of grassroots governments in accomplishing their tasks

During a major public emergency, grassroots governments face a significant increase in organizational tasks. This requires them to handle multiple tasks simultaneously. To ensure the smooth implementation of each task, the attention of grassroots governments needs to be allocated in multiple threads. During the COVID-19 epidemic, grassroots governments are responsible for completing routine tasks such as sealing off epidemic areas, patrolling, and preserving supplies (74). However, the government’s attention is divided among many different tasks, making it difficult to focus attention and governance resources on key tasks. The second manifestation of dispersed attention is the completion of routine tasks over a prolonged period or the gradual transformation of non-routine tasks into routine ones. This is often due to the monotony of grassroots government work or the lack of external stimuli. Attention allocation is characterized by high persistence and low intensity.

#### 4.3.3 Attention mismatch: selective mandate implementation by grassroots governments

Attention mismatch is the allocation of attention resources in an inappropriate location. According to Simon’s theory of limited rationality in decision-making, attention mismatch or imbalance can be caused by organizations’ limited rationality and information overload. Under the pressure-based system, grassroots governments vary in their implementation of higher authority tasks. Driven by self-interest, they respond positively to policies that benefit them, but are passive and neglectful toward other policies. This creates a mismatch between the priorities of higher authorities and grassroots governments. This mismatch causes them to focus solely on the indicators and neglect the underlying issues, resulting in a result-oriented approach to their tasks. Although the ultimate goal of tasks and indicators is to serve the public interest, the quality of public services often fails to receive sufficient attention from the government during policy implementation. This selective approach to task implementation can result in a mismatch between the allocation of attention by grassroots governments and the public interest.

### 4.4 Reasons for problems with attention allocation in grassroots government

#### 4.4.1 Insufficient resources for attention

Variations in the allocation of government attention can impact the policy agenda process, which in turn affects the formulation and implementation of public policies. As a result, government attention has become a subject of competition among different policy interest groups (75). In routine tasks, higher authorities often assess the performance of grassroots governments using informative and quantitative data and forms (76). This requires the attention resources of grassroots governments to be allocated to data and forms for a long period of time, making it difficult for them to spare time to deal with other matters. Grassroots cadres often have a range of responsibilities, including completing tasks assigned by their superiors in functional departments. This can be in addition to their essential work, and can be layered, multidisciplinary, arbitrary, and repetitive, which can increase their workload.

#### 4.4.2 Untimely diversion in attention

To save attention resources, grassroots governments can use the mode of attention diversion in non-routine tasks. This process requires converting human and material resources, as well as related organizational structures and working modes. Campaign-style governance can often divert the central focus of governance (77), and establishing a non-essential temporary institution which drain the government’s attention resources. It is important to maintain a stable and efficient governance structure. Due to the absence of standards and supervision, grassroots governments often face challenges when implementing attention diversion. These challenges include delays caused by vague systems and inconsistent requirements, as well as delays in withdrawing attention resources due to organizational interests and demands. Additionally, corruption for personal gain is not uncommon, resulting in a serious mismatch of attentional resources.

#### 4.4.3 Differences in the degree to which higher-level tasks attract attention at the grass-roots level

In the Chinese government system, higher-level government can effectively control the lower level of government through task decomposition, indicator control, and result assessment. Grassroots governments tend to prioritize indicators with higher incentives when allocating attention and resources. During COVID-19, the number of confirmed cases has become the primary indicator of the effectiveness of the government’s epidemic prevention and control. As a result, achieving zero-cases has become a priority for grassroots governments and has received increased attentional resources. These tasks can be easily quantified and endorsed by superiors, which will easily lead to an over-reliance on quantitative indicators by grassroots governments, resulting in inappropriate and excessive anti-epidemic measures and causing inconvenience to people’s daily lives (78).

## 5 Conclusion and discussion

### 5.1 Key results

This paper proposes a model of attention allocation in situations where the government faces routine and non-routine tasks. Attention allocation is a crucial mediating variable that affects government behavior. The related studies have been reviewed to support this proposal. In routine tasks, the government should exhibit low-intensity attention with high persistence. It is recommended to invest in persistent and stable behavior for routine tasks while allocating fewer resources and allowing for variability in the implementation process to superiors. In non-routine tasks, the government’s focus is characterized by high-intensity, low-persistence attention. The government will primarily adapt to variable and high-intensity attention allocation through campaign governance (79). This involves clustering resources to better implement superior tasks, with attention fading quickly after the goal is completed within a short period of time. The behavior is not continuous, representing a flexible attention-diversion mode.

In this framework, task contexts are independent variables that influence the allocation of government attention. Government behavior, such as the allocation of resources, continuity, and policy implementation, are dependent variables. The study found that the intensity of grassroots governments’ attention to non-routine tasks decreases after the gradual normalization of COVID-19 prevention and control. The model demonstrates that grassroots governments facing the challenge of having less power and more work, optimize their attentional resources through three modes: attentional allocation in routine tasks, attentional allocation model in non-routine tasks, and routinization model through attention diversion. This response logic can result in high efficiency but low effectiveness. Relevant research has revealed the complexity of the government’s attention allocation. Excessive investment can either be the root cause of resource waste or a “manifestation” of institutional flaws. Grassroots officials may shift their attention to tasks that are “easy to quantify” or “visible to superiors,” resulting in Goal Displacement (80). In order to cope with inspections by higher authorities, grassroots governments have devoted excessive energy to organizing archival materials, while neglecting the actual needs of people’s livelihood (81). Phenomena such as the “attention trap” or the “paradox of excessive attention” should be taken seriously.

Therefore, it is important to study the allocation of government attention in order to understand the logic behind the behavior of grassroots governments. This includes exploring the dilemmas of attention fatigue and attention mismatch. The findings of this study can provide theoretical support for grassroots governments to conduct attention allocation more effectively and rationally, thus contributing to the modernization of grassroots governments’ governance capacity and governance system.

In practice, the focus of government attention is mainly determined by the context in which it operates (82). Attention allocation usually remains stable in normal situations, but crises can disrupt the balance of the existing policy system and awaken government attention (83). The attention triggered by a crisis requires an effective response from the political system, and attention allocation is the fundamental way to respond to the crisis (84). Because in a normal state, a highly stable management system will have characteristics such as regular goals, hierarchical control, clear rights and responsibilities, and fixed control. And crisis situations can cause local officials to face more issues such as shifting attention allocation, balancing multiple goals, and conflicting rights and responsibilities (85). The attention dissemination within the Chinese system usually focuses on vertical transmission, and political and administrative affairs are often guided by superiors to subordinate actions (86). Faced with various challenges in crisis, the central government or higher-level government guides the attention allocation of grassroots governments through institutionalized mechanisms such as power authority mechanisms (such as highly valued leadership), incentive mechanisms (such as promotion and accountability), and resource allocation mechanisms, effectively promoting the transformation of the administrative system from “multitasking” in normal times to “crisis management” (87).

Of course, the framework also has shortcomings: the corresponding allocation of attention in a single task situation is only an ideal result. In reality, several tasks are intertwined and the allocation of attention is the result of the joint action of several factors. Regarding the Wuhan epidemic prevention, excessive formalism in some areas has depleted the government’s attention resources and resulted in high allocation toward routine tasks (88). Therefore, the attention allocation model needs to be adapted to the actual situation.

### 5.2 Policy recommendations

#### 5.2.1 Promote attention expansion

To address the limited attention resources, grassroots governments should expand their focus. To manage the excessive number of matters that consume attention resources, higher-level governments should assign tasks effectively and grassroots governments should integrate their work reasonably. To expand the attention of grassroots governments, we should first guide the participation of diverse subjects in grassroots governance (89). Secondly, higher-level governments should take the initiative to delegate power to the grassroots (90) and flexibly deal with the procedure of approving and instructing resource allocation in different situations.

To achieve effective integration of grassroots governments, it is necessary to strengthen grid-based and precise management (91), clarify the responsibilities of grassroots governments, and achieve unity of power and responsibility. It is important to regulate the scope of law enforcement and avoid unlimited departmental responsibilities that exacerbate the scarcity of attention (92). Secondly, grassroots governments should focus on their work and prioritize efficiently allocating attention and resources.

#### 5.2.2 Establishing an institutionalized pathway for attention diversion

To tackle the issue of delayed attention diversion, it is crucial to establish a formalized process for attention diversion. During major crises, it is important to rationalize the allocation of attention resources within the government and strengthen the responsibility of grassroots governments for territorial management. This will enable grassroots governments to allocate attention resources independently, gather resources and concentrate attention when non-routine tasks arise.

To establish an institutionalized approach, grassroots governments must learn from their experiences in handling non-routine tasks. This can be achieved through institutional arrangements that reduce the feeling of helplessness when faced with unexpected challenges. Additionally, such arrangements can strengthen supervision and management, allowing for the timely withdrawal of attention resources once non-routine tasks are completed.

#### 5.2.3 Improving incentives for higher-level assignments

To achieve an orderly allocation of attention, we stimulate the vitality of grassroots governments by scientifically determining indicators and conducting performance assessments. The government’s principal-agent relationship is often hindered by the asymmetry of information (93). To address this problem, it is crucial to establish a monitoring mechanism, higher levels of government should enhance their supervision of key matters to prevent any attention mismatches.

### 5.3 Limitations

This study attempted to propose attention allocation for governments facing both normal and abnormal task scenarios model. Firstly, although the attention allocation model is a perspective for interpreting grassroots government decisions and behaviors, it is not the only one. Secondly, government decisions and actions in the real world are undoubtedly very complex and never have simple explanations. Attention and its allocation are just one of these complex influencing factors. Political pressure, resource constraints, or personal leadership styles can all affect it to varying degrees, in different task contexts, or under other conditions. In addition, the same applies to psychological, emotional, and attitudinal factors. Thirdly, the allocation of attention corresponding to a single task scenario is only an ideal division. In reality, the two tasks are intertwined, and the allocation of attention is also the result of the interaction of various factors. In the theoretical test taking COVID-19 as an example, we found that the model still needs to be discussed. Formalism in some regions consumes government attention resources and shows a high distribution state in conventional tasks. Therefore, the attention distribution model needs to be adjusted according to the actual situation, and the model also needs to be improved by subsequent research. Finally, this study only focuses on the government’s attention distribution in the abnormal situation, especially in a special crisis such as COVID-19. While the COVID-19 provides a unique context to study government behavior, it may not be representative of normal operating conditions. These findings may lean toward crisis management rather than daily management. Considering the above, further in-depth exploration is needed.

## Data Availability

The original contributions presented in the study are included in the article/supplementary material, further inquiries can be directed to the corresponding authors.
